# Dr. Frank H. Potter

**Published:** 1886-12

**Authors:** 


Dr. Frank H. Potter.—Dr. F. H. Potter, of this city, left some time since to continue his studies in diseases of the throat and nose with Dr. Shurly, of Detroit. He will have there a most excellent opportunity to learn this branch, which will be his chosen specialty. The doctor has already had opportunity to study this department at home and in Europe, with the addition of several month's training under this celebrated laryn. gologist, he will be well qualified, and find in Buffalo, where he has many friends, both in and out of the profession, a good field for his labors.

